# Transmission indices and microfilariae prevalence in human population prior to mass drug administration with ivermectin and albendazole in the Gomoa District of Ghana

**DOI:** 10.1186/s13071-015-1105-x

**Published:** 2015-10-26

**Authors:** Fred Aboagye-Antwi, Bethel Kwansa-Bentum, Samuel K. Dadzie, Collins K. Ahorlu, Maxwell A. Appawu, John Gyapong, Michael David Wilson, Daniel Adjei Boakye

**Affiliations:** Noguchi Memorial Institute for Medical Research, University of Ghana, Accra, Ghana; Department of Animal Biology and Conservation Science, University of Ghana, Accra, Ghana; School of Public Health, University of Ghana, Accra, Ghana

**Keywords:** Perceptions, Baseline indices, Human microfilariae, *Anopheles* species, Transmission, Ghana

## Abstract

**Background:**

The Lymphatic Filariasis Elimination Programme in Ghana involves annual mass drug administration (MDA) of ivermectin and albendazole to persons living in endemic areas. This is repeated annually for 4–6 years to span across the reproductive lifespan of adult worms. In order to stimulate participation of community members in the MDA programme, this study was carried out to understand local views on transmission, management and prevention of the disease. The study also presents baseline transmission indices and microfilariae prevalence in the human population in eight endemic communities of coastal Ghana prior to the MDA.

**Methods:**

A descriptive survey was carried out to explore perceptions on causes, treatment and prevention of lymphatic filariasis. Perceptions on community participation in disease control programmes were also assessed. After participants were selected by cluster sampling and 100 μl of blood sampled from each individual and examined for mf microfilariae. A similar volume of blood was used to determine the presence of circulating filarial antigen. Mosquitoes were collected simultaneously at all sites by human landing catches for 4 days per month over a six-month period. All *Anopheles* mosquitoes were dissected and examined for the larval stages of the parasite following which molecular identification of both vector and parasite was done.

**Results:**

Eight hundred and four persons were interviewed, of which 284 (32.9 %; CI 31.1–34.5) acknowledged elephantiasis and hydrocoele as health related issues in the communities. Thirty-three people (3.8 %; CI 2.1–5.5) thought sleeping under bed net could help prevent elephantiasis. Microfilariae prevalence was 4.6 % (43/941) whiles 8.7 % (75/861) were positive for circulating filarial antigen. A total of 17,784 mosquitoes were collected, majority (55.8 %) of which were *Anopheles* followed by *Culex* species (40 %). Monthly biting rates ranged between 311 and 6116 bites/person for all the eight communities together. Annual transmission potential values for *An. gambiae* s.s. and *An. funestus* were 311.35 and 153.50 respectively.

**Conclusion:**

Even though the highest mf density among inhabitants was recorded in a community that had the lowest *Anopheles* density with *Culex* species constituting 95 % of all mosquitoes collected, *Anopheles gambiae* s.s. and *An. funestus* remained the main vectors.

## Background

Lymphatic filariasis (LF) is a disease that can lead to elephantiasis and hydrocoele as the main clinical manifestations. About 120 million people in 83 countries in the tropical and subtropical regions of the world are infected, with an estimated 1.34 billion people at risk [1] (WHO 2010–2020). The disease is caused by three species of filarial worms namely *Wuchereria bancrofti*, *Brugia timori* and *B. malayi*. Among them, *W. bancrofti* causes over 90 % of all LF cases [2] (WHO internet May 2015). Lymphatic filariasis has been identified as the second leading causes of permanent and long-term disability in the world [3] (WHO 1995). The disease is rarely fatal, but clinical manifestations carry grave personal and sociocultural consequences for those affected and their immediate family members. The disease can result in sexual dysfunction, divorce and victims are often not suitable for marriage [4–10] (Gyapong et al., 1996; Gyapong et al., 1996; Gyapong et al., 2000; Dreyer et al., 1997; Ramaiah et al., 1997; 3–9 Coreil et al., 1998; Ahorlu et al., 1999). The disease also negatively impacts on the work output of affected individuals and victims are often subjects of public ridicule [11] (Dunyo et al., 1996).

The disease has been targeted for elimination by the World Health Organization (WHO) through yearly mass drug administration (MDA) of ivermectin and albendazole or diethylcarbamazine to inhabitants of endemic communities [12] (Turner). The drugs are usually given in single doses continuously for 4-6 years, until adult worms have reached the end of their reproductive lifespan. In situations where coverage is low, MDA must be extended in order to interrupt transmission [13] (WHO 2011, Geneva). The elimination strategy is based on the postulation that should microfilarial reservoir in the human host be reduced below a certain threshold, transmission of *Wuchereria bancrofti* by anopheline vectors could be interrupted, a phenomenon known as *facilitation* [14–16] (Weber 1991; Southgate & Bryan 1992; Bockarie et al., 1998). For example, findings from Papua New Guinea (PNG) showed that transmission by *Anopheles punctulatus* was virtually stopped after a year of treatment, although the frequency of microfilariae (mf) carriers in the human population ranged from 10.5–52.7 % [16] (Bockarie et al., 1998). However, it may not be practicable to generalise this PNG observation worldwide. In the West African sub region, several species of *Anopheles*, which are vectors of bancroftian LF occur in sympatry, contrary to the situation in PNG where the disease was transmitted by only one vector species [17] deSouza et alThe threshold of mf frequency needed for elimination of anopheline-transmitted *W. bancrofti* LF may differ from one vector species to another and from one community to another. Additionally, the co-existence of *W. bancrofti* and *Plasmodium falciparum* in most LF endemic communities has led to an interesting relationships being developed between these two parasites. One parasite tends to dominate in prevalence/importance over the other and vice versa at certain points in time during the year, despite sharing similar *Anopheles* vectors and environmental factors that support their survival and multiplication in West Africa [18] (Kelly-Hope et al. 2006). Variations in both human infection burden and the associated environmental and entomological factors influencing the transmission of both diseases may account for the prevalence patterns exhibited, which could in turn affect local LF control efforts.

Many well-meaning and carefully planned control programmes have failed to achieve their desired targets because these programmes have often overlooked the role of community members in those endemic areas [19] (Wynd et al., 2007). It has been observed that views and behaviour of people towards an illness either enhance or interfere with the effectiveness of control measures [20] (Bentum). In addition, participation in control efforts may not be optimum if the communities perceive lymphatic filariasis as a less important health problem [10] (Ahorlu et al.). Since the mainstay for the control programme is not morbidity control but transmission blockage through chemotherapy, the success of such a strategy depends largely on the level of coverage of the drug administration to the target population. Hence understanding community perceptions about the disease is vital to encouraging maximum participation [19] (Wynd et al., 2007).

In view of this, there is the need to monitor and evaluate the trends in transmission of *W. bancrofti* by *Anopheles* species and the human microfilaria densities in the sub-region before, during and after MDA with ivermectin and albendazole to ensure successful LF elimination. The Ministry of Health of the Republic of Ghana initiated its elimination programme in line with WHO recommendation in the year 2000. To complement their efforts towards the elimination of LF, the Noguchi Memorial Institute for Medical Research (Ghana) established sentinel sites in one of the endemic districts in southern Ghana to monitor the national control programme through parasitological and entomological surveys. The resulting data from this monitoring activity were fed into the programme as a means of enhancing its implementation at community level. The study herein reported was conducted to assess the views and perceptions of persons, as well as document vector transmission indices and mf prevalence in the human population in eight endemic communities in the Gomoa district of Ghana.

## Methods

### Study sites and design

**Fig. 1:**
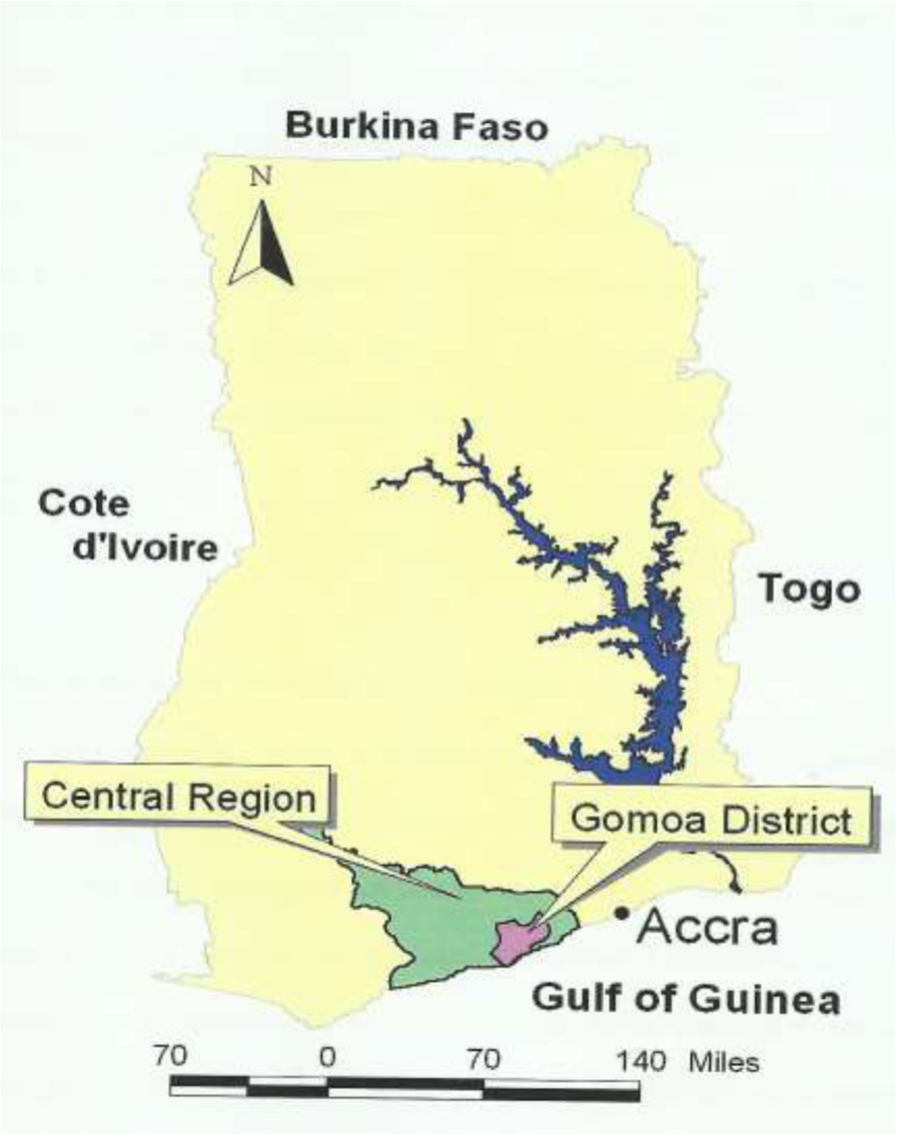
Map of Ghana showing Central Region and the location of Gomoa District where the study was conducted

The study was conducted in eight communities in the Gomoa District of the Central Region of Ghana (Fig. 1), namely Amanful, Ayesuano, Dago, Fawomanye, Hwida, Kyiren, Mampong and Obiri. These communities were identified by the Neglected Tropical Diseases Control Programme of Ghana as LF endemic, and were earmarked for longitudinal community-based mass combination treatment with ivermectin and albendazole. They were also chosen based on their proximity to each other, and the absence of onchocerciasis MDA in these communities. All eight communities lie between Latitude 5° 24’–35’N and Longitude 0° 25’–36’W, which is in the coastal savannah zone located about 50 km west of Accra, the capital city of Ghana. Average annual rainfall ranges between 760 and 1000 mm, with mean annual temperature ranging between 26 and 30 °C. Apart from Ayesuano which majority of the houses made of mud with thatch roof, houses in the remaining communities were predominantly concrete-walled with aluminium sheets as roof. The questionnaire study was done in early 2001. This was followed by the vector studies from July to December of the same year. Parasitological survey on the human population was carried out in early 2002, after which the first in the series of annual MDA was implemented.

### Questionnaire administration

This involved a cross-sectional descriptive study in which questionnaire were administered by enumerators. It explored the views and perceptions of the indigenes on the clinical presentations of lymphatic filariasis (hydrocoele, elephantiasis and acute attacks) its causes, treatment and prevention. Other information sought included knowledge on the commonest ailments and, major health problems identified within the communities, as well as the expectations of community inhabitants of the MDA programme. Respondents were heads of households, their spouses or family representatives. They were selected randomly from a community register with the aid of an SPSS software programme.

### Mass screening for microfilariae in the communities

The baseline community mf prevalence and circulating filarial antigen (CFA) were determined from a cohort of 1200 individuals who were all above 5 years of age. After participants were selected by cluster sampling, written informed consent were obtained. Finger prick blood samples were taken from the study participants between 21.00 and 01.00 h into 100 μl capillary tubes. Each blood sample was dispensed into a 1.5 ml Eppendorf tube containing 900 μl of 3 % acetic acid, mixed thoroughly and transferred into Sedgwick-Rafter counting chamber. This enabled estimation of parasitaemia by counting the number of mf in the chamber using a compound light microscope set at × 100 magnification. Some of the finger-pricked blood were directly used to prepare thin blood smears and stained with Giemsa for identification by microscopy. An additional 100 μl blood samples were collected and used to determine the presence of CFA in the study participants using BinaxNOW® Filariasis Immunochromatographic test (ICT) kit.

### Collection, identification and dissection of mosquitoes

Each village was divided into four sections and a house randomly selected from each section for night collection of mosquitoes using the human-landing catch (HLC) method. Mosquitoes were collected from 18.00–06.00 h Greenwich Mean Time (GMT) for each night’s collection. Four night-collections were carried out each month for six consecutive months (July-December), spanning both the rainy and dry seasons for each of the study sites. Four volunteers were trained as vector collectors for each village, with two doing the collection for six-hour period (18.00–00.00 and 00.00–06.00) at a time.

Collected mosquitoes were morphologically identified as *Anopheles gambiae* s.l., *An. funestus* group, *An. pharoensis*, *An. coustani, Aedes* species or *Culex* species using entomological keys [21, 22] (Gillies & de Meillon 1968; Gillies& Cotzee 1987). Dissections proceeded by first separating each mosquito carcass into head, thorax and abdomen, and subsequently dissecting each part in a drop of normal physiological saline. Each dissected part was microscopically examined for infections with larval stages of *W. bancrofti.* Parasite larval stages (L_1_, L_2_ and L_3_) found in the dissected *Anopheles* carcasses were recorded to estimate entomological indices such as infectivity rate and transmission potential. Infected and uninfected mosquito carcasses were individually transferred into 1.5 ml Eppendorf tubes containing isopropanol and kept at -20 °C until ready for molecular identification.

### Molecular identification of *Anopheles* mosquito species

Slightly modified established protocols were used in the molecular identifications of *An. gambiae* s.l. and *An. funestus* [23, 24] (Collins et al., Scott e al). Genomic DNA was extracted from the carcasses of mosquitoes after homogenizing with sterile plastic pestles in 100 μl bender buffer. The homogenate was incubated at 65 °C for 30 min, followed by the addition of 125 μl of 100 % phenol. The mixture was vortexed and centrifuged at 14,000 rpm for 10 min using Eppendorf® Centrifuge 5415 C. The supernatant was transferred into new 1.5 ml Eppendorf tube. Two hundred and fifty microlitres of pre-chilled absolute ethanol and 10 μl of 8 M potassium acetate were added to the supernatant. This was incubated at -40 °C for an hour, spun at 10,000 rpm for 10 min and supernatant poured off. The pellet was then rinsed with 200 μl of 70 % ethanol, spun at 10,000 rpm for 5 min, and supernatant discarded. The pellet was dried and re-dissolved in 50 μl Tris EDTA buffer with RNAse and kept at 4 °C until ready for PCR. Each PCR reaction mixture of 25 μl contained 1× PCR buffer (Sigma, USA), 200 μM each of the four deoxyribonucleotide triphosphates (dNTPs), 10 μM each of the oligonucleotide primers (Table 1), and 0.125 units of Taq Polymerase enzyme (Sigma, USA). One microlitre of the genomic DNA was used as template for the amplification reaction.

**Table 1:** Oligonucleotide primer sequences and PCR reaction conditions for identification of *Anopheles* species and *Wuchereria bancrofti*

Primer for species ID	Sequence 5’ ⇒ 3’	PCR product size (bp)	PCR conditions
*Anopheles gambiae* s.l. species			
Universal primer	GTGTGCCCCTTCCTCGATGT	468	93 °C 3’ followed by 35 cycles (93 °C 30”; 50 °C 30”; 72 °C 1’); 93 °C 30”; 50 °C 30”; 72 °C 10’
*Anopheles gambiae s.s.*	CTGGTTTGGTCGGCACGTTT	390	
*Anopheles merus/ melax*	TGACCAACCCACTCCCTTGA	464	
*Anopheles arabiensis*	AAGTGTCCTTCTCCATCCTA	315	
*Anopheles quadrianulatus*	CAGACCAAGATGGTTAGTAT	153	
*Anopheles funestus* s.l. species			
Universal primer	TGTGAACTGCAGGACACAT		30 cycles (94 °C 30”; 40 °C 30”; 72 °C 30”); 72 °C 10’
*Anopheles funestus* s.s.	GCATCGATGGGTTAATCATG	460	
*Anopheles vaneedeni*	TGTCGACTTGGTAGCCGAAC	555	
*Anopheles rivulorum*	CAAGCCGTTCGACCCTGATT	400	
*Anopheles parensis*	TGCGGTCCCAAGCTAGGTTC	235	
*Anopheles leesoni*	TACACGGGCGCCATGTAGTT	146	
*Wuchereria bancrofti*			
*NV-1*	CGTGATGGCATCAAAGTAGCG	188	94 °C 3’; followed by 35 cycles (94 °C 1’; 55 °C 1’; 72 °C 2’); 94 °C 1’; 55 °C 1’; 72 °C 10”
*NV-2*	CCCTCACTTACCATAAGACAAC	188	

After DNA amplification, the PCR products were electrophoresed in 2 % agarose gel containing 0.5 μg/ml ethidium bromide in 1× TAE buffer using a mini gel system (BIORAD USA). Eight microlitres of each sample was mixed with 1 μl of 5× Orange G loading dye and loaded into a well of the gel. The voltage of the power unit was set at 100 V and run for an hour. A photograph of the gel was obtained using a UV trans-illuminator (UPC, USA) and a Polaroid camera of film type 667 (Polaroid, USA). The sizes of the PCR products were estimated by comparison with the mobility of a 100 base pair molecular weight size marker (Sigma).

### Molecular identification of *Wuchereria bancrofti* larvae in mosquito vectors

Using the carcass of infected mosquitoes, filarial parasites were identified molecularly by following established protocol with a few modifications [25] (Ramzy et al.). DNeasy Tissue Kit (QIAGEN Inc., USA) was used in the extraction of parasite DNA from animal tissues by following the manufacturer’s recommended protocol. After DNA extraction, aliquots of 5 μl of filarial DNA extract from the mosquitoes were used as templates for the amplification reaction. The PCR assay was performed using two specific oligonucleotide primers, NV-1 and NV-2 [Table 1] following which the products were electrophoresed in 2 % agarose gel as previously described.

### Community entry and ethical consideration

Prior to data collection, the purpose for the study was explained to the residents through community durbars. Written informed consent were obtained from participants after guaranteeing their anonymity and informing them of their rights to freely discontinue participation in the study any time they felt so without any repercussions. The study was reviewed and approved by the Institutional Review Board (IRB) of the Noguchi Memorial Institute for Medical Research and the Ethical Committee of the World Health Organization, Geneva. The first in the series of the yearly MDA with ivermectin and albendazole started immediately after mass screening of blood samples of participants for the microfilaria.

### Data analyses

The questionnaire data were analysed using EpiInfo version 6 and SPSS 10.1 (SPSS Inc, Cary, USA) software package. All data generated were assessed for normality and descriptive statistics such as frequencies and mean (±SD) values of infection prevalence were calculated. Differences in proportions were calculated by Chi-square, whiles confidence interval (CI) was calculated by one-way ANOVA with significance level at 0.05. Entomological and parasitological data were entered into Microsoft Excel spread sheet. Using the formulae for calculating entomological indices (Figs. 9 and 10), programmes were developed in Microsoft Access to compute the transmission indices of the dissected mosquitoes. Again, Microsoft Access was used to calculate the geometric mean intensity of mf in the human population. With *p-*value set at 0.05, one-way ANOVA was used to test for significant differences in filarial parasite load among the various *Anopheles* species.

## Results

### Demographic characteristics

A total of 6921 inhabitants of which 53.4 % were males were enumerated from the eight communities with their age distribution ranging from day zero to 98 years (mean = 26.6; median = 21). Dago was the most populated, constituting 60.3 % of the total. In all, 864 households were enumerated, which translates into an average of 8 persons/ household. A total of 182 (21.1 %; CI 19.9–22.8) respondents were into fishing, 123 (14.2 %; CI 12.6–16.2) were into farming and 83 (9.6 %; CI 8.1–11.4) into petty trading. One hundred and sixty-five (19.1 %; 17.9–20.6) were unemployed.

### Perceptions on general health issues

Community members accessed health care from three main sources; 430 (49.8 %; CI 48.8–51.1) visit clinic/hospital, 194 (22.4 %; CI 21.2–21.0) make use of herbal medicine either purchased from shop or home-made, with 188 (21.8 %; CI 20.4–23.4) visiting a licensed chemical store for an over-the-counter medicine. A total of 331 (38.3 %; CI 36.5–40.2) and 101 (11.7 %; CI 10.7–13.1) mentioned malaria/fever and diarrhoea among the various ailments that are common in the communities. Acute attacks (adenolymphangitis) were seen as part of the fever complex which were used to refer to the many conditions described as ‘feeling unwell’ in the communities. Thirteen (1.5 %; CI 0.8–2.0) respondents reportedly slept in bed nets the night preceding the interview, with only 2 (0.2 %) of the nets treated with insecticide. Six hundred and thirty-one (73 %; CI 70.2–75.1) of respondents blamed their lack of interest in participating in the control programme on not seeing any immediate direct benefit they could gain from the control programme.

### Perceptions on elephantiasis

**Fig. 2:**
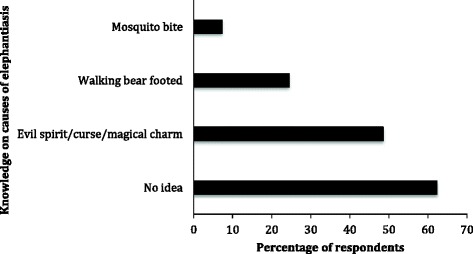
Respondents’ perception on causes of elephantiasis. More than half of respondents had no idea on what causes elephantiasis. For those who had ideas, less than 10 % mentioned mosquito bite as one of the ways the elephantiasis could spread. Multiple answers were allowed

Two hundred and eighty-four (32.9 %; CI 31.1–34.5) respondents said elephantiasis, which is known locally as “gyepim/pefur” was a common ailment in the communities. Evil spirits and mosquito bites were mentioned as the causes of elephantiasis (Fig. 2). Available health seeking options for elephantiasis treatments showed parity between herbal medicine (either home-made or by traditional healers) and orthodox medicine (from clinic/ hospital), contributing 164 (19.0 %; CI 18.2–20.2) and 165 (19.1 %; CI 18.3–20.5) respectively. Forty-eight (5.6 %; CI 4.8–6.4) also self-medicate through buying drugs from street vendors, while 11 (1.3 %; CI 0.5–2.1) prescribed smearing local gin (Akpeteshie) on the swollen limbs to manage elephantiasis. On preventive measures, 215 (24.9 %; CI 22.9–26.7) mentioned that adhering to medical advice provided by health personnel could help. Thirty-three (3.8 %; CI 2.1–5.5) and 25 (2.9 %; CI 1.7–4.9) thought that sleeping under bed net and having access to good drinking water respectively could prevent elephantiasis. Other less prominent preventive options cited by respondents included eating balanced diet, reduction in hard work and regular taking of medicines particularly herbal preparations [2 (0.2 %; CI 0.05–0.35)]. Challenges faced by elephantiasis patients included impaired ability to walk for long period of time [191 (22.1 %; CI 21.6–22.7)], being less productive [79 (9.1 %; CI 8.2–9.9)] as well as patients being stigmatised [51 (5.9 %; CI 4.9–6.7)]. Other challenges include their inability to participate in sports and other social activities.

### Perceptions on hydrocoele

**Fig. 3:**
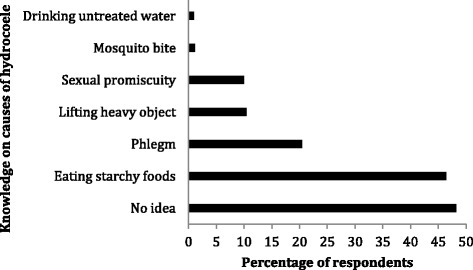
Respondents’ perception on causes of hydrocoele. Almost half of respondents had no idea on what causes hydrocele. About 10 % attributed lifting heavy objects (without wearing the right clothing) as the cause of hydrocoele. Perhaps those with this information think hernia is synonymous with hydrocoele. However, less than 2 % mentioned mosquito bite as one of the ways hydrocoele could spread. Multiple answers were allowed

**Fig. 4:**
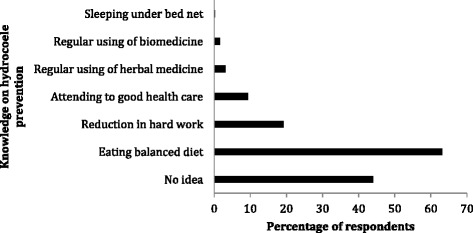
Respondents’ perception on hydrocoele prevention. About 44 % of respondents had no idea on how hydrocoele could be prevented. A meagre 0.2 % said sleeping under bed net could prevent the transmission of the pathogen that causes hydrocoele. Multiple answers were allowed

As many as 218 (25.2 %; CI 24–26.4) respondents recognised hydrocoele, which is locally called”etow” as a common ailment in the study communities. Some of the known causes of hydrocoele according to respondents include engaging in hard work such as lifting heavy objects [90 (10.4 %; CI 9.2–11.8)] and mosquito bites [10 (1.2 %; CI 0.7–1.7)] (Fig. 3). Two hundred and twenty-four (25.9 %; CI 24.7–27.1) respondents disclosed that hydrocoele could be prevented, but only 2 (0.2 %; CI 0.05–0.35) mentioned sleeping in bed-net as one of the preventive methods (Fig. 4). Two hundred and thirty-five (27.2 %; CI 26–28.4) acknowledged that hydrocoele affect the patient’s ability to walk properly which reduces productivity. One hundred and sixty-six (19.2 %; CI 17.7–20.8) respondents admitted that hydrocoele patients were stigmatised in the community.

### Prevalence of microfilariae in the study communities

A total of 941 and 861 blood samples were examined by microscopy and immunochromatographic test (ICT) kit respectively. This gave an overall parasite prevalence of 4.6 % (*N = 43*) by microscopy examinations and an antigenaemia of 8.7 % (*N = 75*) on assessment by ICT kits (Table 2). In two of the study communities (Amanful and Kyiren), no prevalence of both mf and circulating filarial antigen was recorded (Table 2). Overall mf density ranged between 0–5010 mf/ml of blood (Table 2), with a significant difference in infection being observed between males 6.9 % (32/464) and females 2.3 % (11/477) [*p* < 0.05].

**Table 2:** Community prevalence of lymphatic filariasis either using microscopy or immunochromatographic test kit

Study community	Microscopy								Immunochromatographic test kit					
	No. screened	Male ♂	Female ♀	No. positive ♂	No. positive ♀	Mf density range	Mean mf density	Mf prevalence (%)	No. screened	Male ♂	Female ♀	No. positive ♂	No. positive ♀	Mf prevalence (%)
Amanful	44	18	26	0	0	0	0	0	44	18	26	0	0	0
Ayesuano	52	34	18	1	0	60	60	1.92	52	34	18	4	0	7.69
Dago	442	216	226	14	7	10–5010	480	4.75	362	171	191	24	13	10.22
Fawomanyo	53	37	16	2	0	30–140	85	3.77	53	37	16	2	0	3.77
Hwida	100	53	47	11	1	10–3230	1118	12	100	53	47	17	5	22
Kyiren	120	41	79	0	0	0	0	0	120	41	79	0	0	0
Mampong	50	22	28	4	3	20–550	207	14	50	22	28	4	3	14
Obiri	80	43	37	0	0	0	0	0	80	43	37	2	1	3.75
TOTAL	941	464	477	32	11	0–5010	243.75	4.57	861	419	442	53	22	8.71

*Mf* microfilariae

### Distribution of mosquito species collected from the study communities

**Fig. 5:**
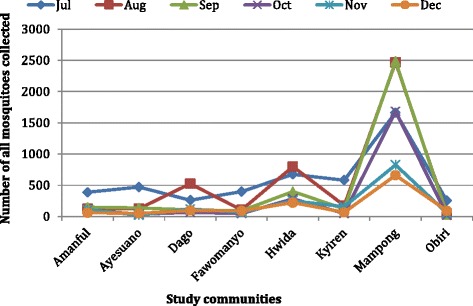
Number of all mosquito species collected from the study communities. A total of 17,784 individual mosquitoes were collected from all eight communities, with Mampong contributing more than half of the mosquitoes. More mosquitoes were caught in July than any other month during the study period

**Fig. 6:**
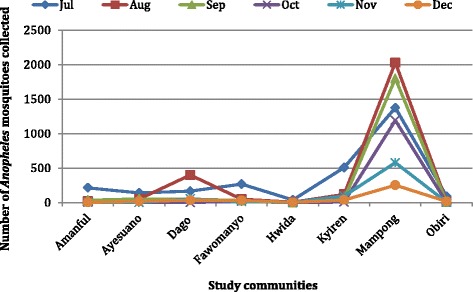
Number of *Anopheles* species collected from the study communities. A total of 9924 (55.8 %) of the mosquitoes were *Anopheles* species, with Mampong alone contributed over 70 % of this number. Forty percent of the mosquitoes caught were *Culex* species, whiles the remaining few were *Mansonia* and *Aedes* species

**Fig. 7:**
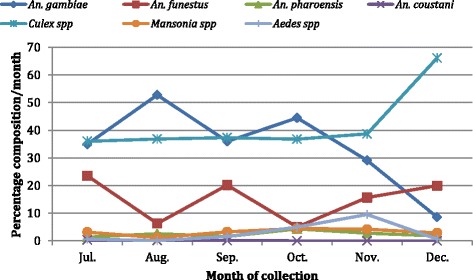
Proportion of the various mosquito species collected according to month. Almost 70 % of the *Anopheles* species were *An. gambiae* s.l., followed by *An. funestus* s.l. These two species of *Anopheles* mosquitoes were found in all the study communities, unlike *An. coustani* which was found in two of the eight sites

**Fig. 8:**
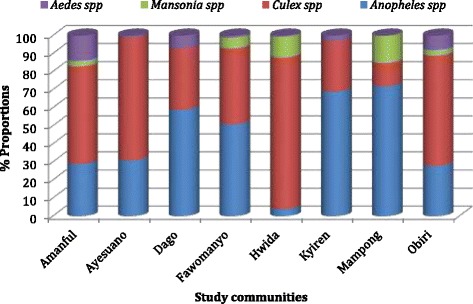
Relative abundance of mosquito species according to study communities. Whereas about 72 % of mosquitoes caught from Mampong were *Anopheles* species, over 84 % of those from Hwida were *Culex* species. Apart from *Mansonia* species which was found in five of the communities, members of the remaining mosquito species were caught in all eight sites

The total number of mosquitoes collected from the eight communities during the study period was 17,784. The highest number of mosquitoes (4686) was recorded in July with the lowest (1330) in December (Fig. 4). An average of 2964 mosquitoes were collected each month, with over 55 % from Mampong (Fig. 5). A total of 9924 (55.8 %) of the mosquitoes were *Anopheles* species of about 72.8 % were collected from Mampong (Fig. 6). The rest were *Culex* spp. (40 %), *Mansonia* spp. (2.2 %) and *Aedes* spp. (2 %). Of the *Anopheles* spp. collected, *An. gambiae* s.l. was the predominant species forming 69 %, followed by *An. funestus* s.l. (26.9 %), *An. pharoensis* (4 %) and *An. coustani* (0.1 %) (Fig. 7). *Anopheles coustani* was found in two of the communities (Amanful and Fawomanyo), whereas *An. pharoensis* occurred in 6 communities. Both *An. gambiae* and *An. funestus* were found in all the study communities. *Anopheles funestus* s.l. was the most collected than any other *Anopheles* spp. In the month of December (Fig. 7). In terms of relative abundance of mosquito species, about 95 % of the mosquito population collected at Hwida was *Culex* species (Fig. 8).

### Transmission indices of *Anopheles* mosquitoes

**Fig. 9:**
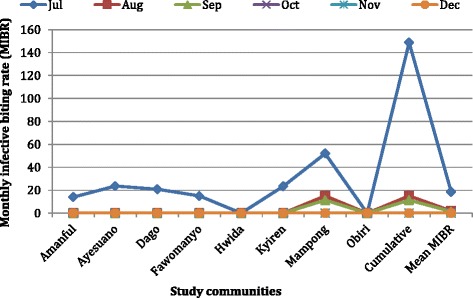
Monthly infective biting rates (MIBR) for the study communities. Monthly infective biting rate (MIBR) = Monthly biting rate (MBR) × Infectivity rate (IR). MBR = man biting rate (mBR) × 30; mBR, which also represents number of a particular species of mosquito biting human per given time = number of mosquitoes caught/ (number of collectors × number of captures). IR = proportion of a particular species of mosquito carrying *W. bancrofti* L_3_ stage larvae

**Fig. 10:**
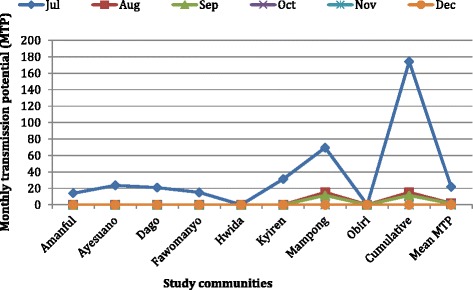
Monthly transmission potential (MTP) for the study communities. Monthly transmission potential (MTP) = Monthly infective biting rate (MIBR) × worm load. MIBR = Monthly biting rate (MBR) × Infectivity rate (IR). MBR = man biting rate (mBR) × 30; mBR, which also represents number of a particular species of mosquito biting human per given time = number of mosquitoes caught/ (number of collectors × number of captures). IR = proportion of a particular species of mosquito carrying *W. bancrofti* L_3_ stage larvae. Worm load = total number of L_3_/ total number of mosquitoes carrying L_3_

The average monthly biting rate of mosquitoes for all the sites was 1115 bites/person and ranged from 311 bites/person at Obiri to 6116 bites/person at Mampong. Of the 9924 *Anopheles* mosquitoes examined, 67 were infected (harbouring any developmental stage (s) of the parasite [L_1_, L_2_ or L_3_ larvae]) of which 31 were infective (harbouring only the third larval stage [L_3_]). The total numbers of L_1_, L_2_ and L_3_ harvested were 33, 2 and 35 respectively. *Anopheles gambiae* s.l. constituted 65.7 % and 51.6 % of infected and infective anopheline mosquitoes respectively, and harboured 47 of the 70 parasite’s life stages found. Monthly infective biting rate (MIBR) of *Anopheles* mosquitoes ranged between 0–52 infective bites/person (Fig. 9). The highest monthly transmission potential (MTP) of 47.8 % was recorded at Mampong, with the observed trend closely mimicking that of the MIBR (Fig. 10). Most transmission occurred in July, with anophelines from Mampong accounting for 73 % of the total number of *Anopheles* mosquitoes biting humans at any given time. Annual infective biting rate (AIBR) for *An. gambiae* and *An. funestus* were 119.75 and 113.70 respectively. The annual transmission potential (ATP) due to *An. gambiae* and *An. funestus* were 311.35 and 153.50 respectively.

### Molecular identification of *Anopheles* mosquitoes and *Wuchereria bancrofti* larvae

Only *An. gambiae* s.l. and *An. funestus* s.l. mosquitoes were identified at the molecular level. A total of 584 *Anopheles* mosquitoes, consisting of 67 infected and 517 randomly selected *Anopheles* mosquitoes (Table 3) were analysed. Of these, 413 (93.9 %) were identified as *An. gambiae* s.s., 114 (79.2 %) as *An. funestus* s.s. and 19 (13.3 %) as *An. leesoni*. Out of the 67 infected mosquitoes (identified by microscopy), molecular identification confirmed 61 of them as *W. bancrofti*.

**Table 3:** Number of mosquitoes identified by polymerase chain reaction

*Anopheles* species	Infected (infective)	Uninfected	Total examined	Successfully identified
*An. gambiae* s.l.	44 (16)	396	440	413 (93.9)
*An. funestus*	23 (15)	121	144	133 (92.4)
Total	67 (31)	517	584	546 (93.5)

## Discussion

The study reported here was in two parts; the first was to shed light on local views on causes of transmission, management (or treatment), control and prevention of lymphatic filariasis in order to factor in such local experiences and perceptions into control efforts towards augmenting the MDA for successful LF control. Disease control programmes in many developing countries are often unsuccessful or unsustainable [19] (Wynd et al. 2007). This is attributed to the inappropriateness of strategies pursued for the local community or the incompatibility of such strategies with local perceptions on aetiology, transmission, treatment and prevention of that disease [4, 10, 26]. (Gyapong et al., 1996; Ahorlu et al., 1999; Ramaiah et al., 2014).

The findings showed that residents were aware of some of the clinical manifestations of lymphatic filariasis that were being described with local names or terms. Indeed, elephantiasis and hydrocoele were identified as health problems in the communities. To the respondents, fever can cause anything including headache, swollen legs, chills and rigors. It was therefore not surprising to have fever mentioned as one of the causes of elephantiasis. The study revealed majority of the individuals living in endemic areas not to be aware of how the pathogen gets transmitted. This could be due to lack of adequate information on the cause of the disease among the study communities. It was insightful observing that majority of the respondents identified hydrocoele, one of the clinical signs of lymphatic filariasis as a common health problem. This acknowledgement could be exploited to boost residents’ interest in getting better informed on the disease towards active participation in the control activities, such as encouraging hydrocoele patients to seek medical care by undergoing corrective surgical operations to remove compromised tunica vaginalis tissues [27] (Addis and Bradley, 2007). Majority of inhabitants in the study communities lacked the motivation to participate fully in the control programme mainly because they did not see any immediate direct benefit to be derived from the control programme. According to the respondents, as long as one does not display any clinical sign of LF, there is no way one could be harbouring the filarial worms. This implies that there is the likelihood of the disease progressing on to clinical disease conditions among such respondents. There is thus the need to highlight the importance of vigorous health educational campaigns in endemic communities prior to the start of disease control programmes in order to ensure community compliance and the eventual success of the programmes.

The second part of the study was designed to document vector transmission indices and mf prevalence in the human population before the commencement of the MDA programme. This data provides the basis for assessing the success or otherwise of the programme, as well as offering insight into the role of the different but sympatric *Anopheles* species in the transmission of lymphatic filariasis in Ghana. An endemic community is said to have a low mf density when the density of circulating mf is less than 200 mf per ml of blood, an amount which cannot be detected in a significant number of instances when commonly used blood sampling techniques are employed [28] (Southgate 1992). By this definition, three of the eight communities (Hwida, Dago and Mampong) had high mf density. Reasons that could be adduced for the mf prevalence and intensities in these study populations include occupational activities of inhabitants [11, 29, 30] (Dunyo et al., 1996; Dzodzomenyo et al., 1999; Appawu et al., 2001). Farming and fishing activities expose them to bites of anopheline vectors, which are known to be the main vectors of lymphatic filariasis in Ghana [11, 30] (Dunyo et al., Appawu et al., 2001). Studies on the relationship between mf density in blood meals and the percentage of *Anopheles* mosquitoes that ingest mf have not provided consistent results [31–35] (Boakye et al., 2004; Coulibaly et al., 2013; McGreevy et al., 1982; Bryan et al., 1990; Bryan & Southgate). Although many of the mf ingested by *Anopheles* mosquitoes are reportedly damaged by the mosquito’s foregut armature, the proportion of mf destroyed is independent of the number of mf ingested and varies between members of the *An. gambiae* complex and *An. funestus* [15, 36] (Southgate & Bryan 1992; Amuzu et al., 2010).

Various species of the *Anopheles* mosquitoes have been identified as important vectors of lymphatic filariasis in Ghana. For example, *An. melas* and *An. funestus* are important in the coastal Western Region [11] (Dunyo et al.). In the Central Region, *An. gambiae* s.s. has been reported to be the most important vector followed by *An. funestus* and *An. pharoensis* [29] (Dzodzomenyo et al.,), whiles in the Upper East Region *An. arabiensis* was found to be the major vector [30] (Appawu et al.). In this study, *An. gambiae* s.s. and *An. funestus* were observed as vectors for the parasite, supporting the findings of Dzodzomenyo and colleagues [29]. Collection of mosquitoes for this study was done from July to December; the first three months fall within the rainy season whereas the last three are part of the dry season of Ghana. While *An. gambiae* s.l. population took a dip in the dry season (December), *An. funestus* was high during this same period. This underscores the ability of *An. funestus* to cope with drought conditions during the dry season, while sustaining parasite transmission [30]. Such an ability of *An. funestus* may be attributed to their ecological flexibility to utilise inundated areas where dry season breeding opportunities exist [30].

There has been no onchocerciasis MDA in these study communities, hence the mf intensity observed was before the introduction of ivermectin and albendazole combination therapy. At this stage of the control programme, one can only be hopeful that after continuous administration of the therapy with a wider coverage area, the disease would be brought under control provided that these *Anopheles* vectors exhibit facilitation. The ATP for *An. gambiae* was about twice that of *An. funestus.* This is as a result of the higher number of *An. gambiae* caught and also found infective than *An. funestus* in the study communities. Studies have shown that it is possible for developing countries to effectively control lymphatic filariasis even with limited funds when cooperative efforts combine with motivation [37, 38] (Sodahlon et al.; Goldman et al.). By this, the community members should be encouraged to see the need to participate in the MDA for optimum coverage size. A study has shown that at low-level parasitaemia (after four rounds of MDA), there was no recovery of infective stage larvae of the parasite, even though the numbers of mosquitoes were low [39] Bentum. It is our hope that with this baseline information, we could effectively assess the impact of MDA on the transmission of the parasite since the same mosquito species were identified to be the vectors.

With *An. gambiae* s.s. and *An. funestus* being the vectors for lymphatic filariasis transmission, we recommend that the use of bed net should not only be encouraged but also added to the filariasis control programme. This will augment disease control efforts in addition to it being promoted for malaria control since both diseases are caused by the common mosquito vector in our West Africa [40] (Njenga et al.). Indeed recent studies have shown the windfall effects of malaria control programmes on lymphatic filariasis transmission [41] (Kelly-Hope et al., 2013), even when LF control has been interrupted [42] (Reimer et al. 2013). Nonetheless, a potential set back to this vector control programme is the emergence of insecticide resistant mosquitoes, which can be overcome by employing appropriate measures such as reviewing the insecticides that are used.

## Conclusion

Community members were aware of some clinical manifestations of lymphatic filariasis. The lack of motivation to participate in the control activities was due to their perception that there is no immediate direct benefit to their lives. *Anopheles gambiae* s.s. and *An. funestus* were the vectors for transmitting the parasite. Since these mosquitoes are also vectors of malaria, we recommend the use of and inclusion of bed net to the filariasis control programme. Intensive health educational campaigns in endemic communities are also needed to encourage community participation in the MDA programme.

## References

[1] WHO. Progress report 2000-2009 and strategic plan 2010-2020 of the global programme to eliminate lymphatic filariasis: halfway towards eliminating lymphatic filariasis. 1. Elephantiasis, Filarial - prevention and control. 2. Elephantiasis, Filarial - epidemiology. 3. Health plans and programs. 4. Program evaluation. I. World Health Organization, 2010. WHO Library Cataloguing-in-Publication Data. Pp1.

[2] WHO: http://www.who.int/lymphatic_filariasis/epidemiology/en/. Accessed on 1st May 2015.

[3] WHO (1995). The world health report 1995: bridging the gaps.

[4] Gyapong JO, Gyapong M, Evans BD, Aikins MK, Adjei S (1996). The economic burden of lymphatic filariasis in northern Ghana. Trop Med Parasitol.

[5] Gyapong M, Gyapong JO, Adjei S, Vlassoff C, Weiss M (1996). Filariasis in northern Ghana: some cultural beliefs and practices and their implications for disease control. Soc Sci Med.

[6] Gyapong M, Gyapong J, Weiss M, Tanner M (2000). The burden of hydrocele on men in Northern Ghana. Acta Trop.

[7] Dreyer G, Noroes J, Addiss D (1997). The silent burden of sexual disability associated with lymphatic filariasis. Acta Trop.

[8] Ramaiah KD, Kumar KNV, Ramu K, Pani SP, Das PK (1997). Functional impairment caused by lymphatic filariasis in rural areas of South India. Trop Med Int Health.

[9] Coreil JJ, Mayard G, Louis-charles J, Addiss D (1998). Filarial elephantiasis among Haitian women: social context and behavioural factors in treatment. Trop Med Int Health.

[10] Ahorlu CK, Dunyo SK, Koram FK, Aagaard-Hansen J, Simonsen PE (1999). Lymphatic filariasis related perceptions and practices on the coast of Ghana: implications for prevention and control. Acta Trop.

[11] Dunyo SK, Appawu M, Nkrumah FK, Baffoe-Wilmot A, Pedersen EM, Simonsen PE (1996). Lymphatic filariasis on the coast of Ghana. Trans R Soc Trop Med Hyg.

[12] Turner J (1997). For WHO or for whom?. Lancet.

[13] WHO (2011). Monitoring and epidemiological assessment of mass drug administration in the Global Programme to Eliminate Lymphatic Filariasis: a manual for national elimination programmes.

[14] Weber RH (1991). Eradication of *Wuchereria bancrofti* through vector control. Trans R Soc Trop Med Hyg.

[15] Southgate BA, Bryan JH (1992). Factors affecting transmission of *Wuchereria bancrofti* by anopheline mosquitoes. 4. facilitation, limitation, proportionality and their epidemiological significance. Trans R Soc Trop Med Hyg.

[16] Bockarie MJ, Alexander ND, Hyun P, Dimber Z, Bockarie F, Ibam E, Alpers MP, Kazura JW (1998). Randomised community-based trial of annual single-dose diethylcarbamazine with or without ivermectin against *Wuchereria bancrofti* infection in human beings and mosquitoes. Lancet.

[17] de Souza DK, Koudou B, Kelly-Hope LA, Wilson MD, Bockarie MJ, Boakye DA (2012). Diversity and transmission competence in lymphatic filariasis vectors in West Africa, and the implications for accelerated elimination of *Anopheles*-transmitted filariasis. Parasit Vectors.

[18] Kelly-Hope LA, Diggle PG, Rowlingson BS, Gyapong JO, Kyelem D, Coleman M, Thomson MC, Obsomer V, Lindsay SW, Hemingway J, Molyneux DH (2006). Negative spatial association between lymphatic filariasis and malaria in West Africa. Trop Med Int Health.

[19] Wynd S, Melrose WD, Durrheim DN, Carrond J, Gyapong M (2007). Understanding the community impact of lymphatic filariasis: a review of the sociocultural literature. Bull World Health Organ.

[20] Kwansa-Bentum B, Ayi I, Suzuki T, Otchere J, Kumagai T, Anyan WK, Asahi H, Akao N, Wilson MD, Boakye DA, Ohta N (2011). Administrative practices of health professionals and use of artesunate-amodiaquine by community members for treating uncomplicated malaria in southern Ghana: implications for artemisinin-based combination therapy deployment. Trop Med Int Health.

[21] Gillies MT, De Meillon B (1968). The Anophelinae of Africa south of Sahara (Ethiopian Zoogeographical Region). S Afr Med J.

[22] Gillies MT, Coetzee M (1987). A supplement to the Anophelinae of Africa south of the Sahara. S Afr Med J..

[23] Collins FH, Mendez MA, Rasmussen MO, Mehaffey PC, Besansky NJ, Finnerty V (1987). A ribosomal RNA gene probe differentiates member species of the *Anopheles gambiae* complex. Am J Trop Med Hyg.

[24] Scott JA, Brogdon WG, Collins FH (1993). Identification of single specimens of the *Anopheles gambiae* complex by the polymerase chain reaction. Am J Trop Med Hyg.

[25] Ramzy RM, Farid HA, Kamal IH, Ibrahim GH, Morsy ZS, Faris R, Weil GJ, Williams SA, Gad AM (1997). A polymerase chain reaction-based assay for detection of *Wuchereria bancrofti* in human blood and *Culex pipiens*. Trans R Soc Trop Med Hyg.

[26] Ramaiah KD, Ottesen EA (2014). Progress and impact of 13 years of the Global Programme to Eliminate Lymphatic Filariasis on reducing the burden of filarial disease. PLoS Negl Trop Dis.

[27] Addis DG, Brady MA (2007). Morbidity management in the Global Programme to Eliminate Lymphatic Filariasis: a review of the scientific literature. Filaria J.

[28] Southgate BA (1992). The significance of low density microfilareamia in the transmission of lymphatic filarial parasites. J Trop Med Hyg.

[29] Dzodzomenyo MD, Dunyo SK, Ahorlu CK, Coker WZ, Appawu MA, Pedersen EM, Simonsen PE (1999). Bancroftian filariasis in an irrigation project community in southern Ghana. Trop Med Int Health.

[30] Appawu MA, Dadzie SK, Baffoe-Wilmot A, Wilson MD (2001). Lymphatic filariasis in Ghana: entomological investigation of transmission dynamics and intensity in communities served by irrigation systems in the Upper East Region of Ghana. Trop Med Int Health.

[31] Boakye DA, Wilson MD, Appawu MA, Gyapong J (2004). Vector competence for *Wuchereria bancrofti* of the *Anopheles* populations in the Bongo District of Ghana. Ann Trop Med Parasitol.

[32] Coulibaly YI, Dembele B, Diallo AA, Kristensen S, Konate S, Dolo H, Dicko I, Sangare MB, Keita F, Boatin BA, Traore AK, Nutman TB, Klion AD, Toure YT, Traore SF (2013). *Wuchereria bancrofti* transmission pattern in southern Mali prior to and following the institution of mass drug administration. Parasit Vectors.

[33] McGreevy PB, Kolstrup N, Tao J, McGreevy MM (1982). Marshall TF deC: Ingestion and development of *Wuchereria bancrofti* in *Culex quinquefasciatus*, *Anopheles gambiae* and *Aedes aegypti* after feeding on humans with varying densities of microfilariae in Tanzania. Trans R Soc Trop Med Hyg.

[34] Bryan JH, McMahon P, Barnes A (1990). Factors affecting transmission of *Wuchereria bancrofti* by anopheline mosquitoes. 3. Uptake and damage to ingested microfilariae by *Anopheles gambiae*, *An arabiensis*, *An. merus* and *An. funestus* in East Africa. Trans R Soc Trop Med Hyg.

[35] Bryan JH, Southgate BA (1988). Factors affecting transmission of *Wuchereria bancrofti* by anopheline mosquitoes. 1. Uptake of microfilariae. Trans R Soc Trop Med Hyg.

[36] Amuzu H, Wilson MD, Boakye DA (2010). Studies of *Anopheles gambiae* s.l. (Diptera: Culicidae) exhibiting different vectorial capacities in lymphatic filariasis transmission in the Gomoa district, Ghana. Parasit Vectors.

[37] Sodahlon YK, Dorkenoo AM, Morgah K, Nabiliou K, Agbo K, Miller R, Datagni M, Seim A, Mathieu E (2013). A success story: Togo is moving toward becoming the first sub-Saharan African nation to eliminate lymphatic filariasis through mass drug administration and countrywide morbidity alleviation. PLoS Negl Trop Dis.

[38] Goldman AS, Guisinger VH, Aikins M, Amarillo MLE, Belizario VY, Garshong B, Gyapong J, Kabali C, Kamal HA, Kanjilal S, Kyelem D, Lizardo J, Malecela M, Mubyazi G, Nitiema PA, Ramzy RMR, Streit TG, Wallace A, Brady MA, Rheingans R, Ottesen EA, Haddix AC (2007). National mass drug administration costs for lymphatic filariasis elimination. PLoS Negl Trop Dis.

[39] Kwansa-Bentum B, Aboagye-Antwi F, Otchere J, Wilson MD, Boakye DA (2014). Implications of low-density microfilariae carriers in *Anopheles* transmission areas: molecular forms of *Anopheles gambiae* and *Anopheles funestus* populations in perspective. Parasit Vectors.

[40] Njenga SM, Mwandawiro CS, Wamae CN, Mukoko DA, Omar AA, Shimada M, Bockarie MJ, Molyneux DH (2011). Sustained reduction in prevalence of lymphatic filariasis infection in spite of missed rounds of mass drug administration in an area under mosquito nets for malaria control. Parasit Vectors.

[41] Kelly-Hope LA, Molyneux DH, Bockarie MJ (2013). Can malaria vector control accelerate the interruption of lymphatic filariasis transmission in Africa; capturing a window of opportunity?. Parasit Vectors.

[42] Reimer LJ, Thomsen EK, Tisch DJ, Henry-Halldin CN, Zimmerman PA, Baea ME, Dagoro H, Susapu M, Hetzel MW, Bockarie MJ, Michael E, Siba PM, Kazura JW (2013). Insecticidal bed nets and filariasis transmission in Papua New Guinea. N Engl J Med.

